# Development of Ti-Coated Ferromagnetic Needle, Adaptable for Ablation Cancer Therapy by High-Frequency Induction Heating

**DOI:** 10.3390/jfb3010163

**Published:** 2012-03-06

**Authors:** Takashi Naohara, Hiromichi Aono, Tsunehiro Maehara, Hideyuki Hirazawa, Shinya Matsutomo, Yuji Watanabe

**Affiliations:** 1 Graduate School of Science and Engineering, Ehime University, Matsuyama 790-8577, Japan; E-Mails: aono.hiromichi.mf@ehime-u.ac.jp (H.A.); maehara.tsunehiro.mg@ehime-u.ac.jp (T.M.); 2 Department of Environmental Materials Engineering, Niihama National College of Technology, Niihama 792-8580, Japan; E-Mail: hirazawa@mat.niihama-nct.ac.jp; 3 Department of Electronic Control Engineering, Niihama National College of Technology, Niihama 792-8580, Japan; E-Mail: shin@ect.niihama-nct.ac.jp; 4 Department of Surgery, Graduate School of Medicine, Ehime University, Toon 791-0295, Japan; E-Mail: yuji@m.ehime-u.ac.jp

**Keywords:** cancer therapy, ablation treatment, high-frequency induction heating, AC magnetic field, shape magnetic anisotropy, magnetic flux direction, biocompatibility

## Abstract

To develop a novel ablation therapy for human solid cancer, the heating properties of a ferromagnetic carbon steel rod and a prototype Ti-coated needle using this carbon steel rod, were investigated in several high-frequency outputs at 300 kHz. In the former, the heating property was drastically different among the three inclination angles (θ = 0°, 45° and 90°) relative to the magnetic flux direction as a result of the shape magnetic anisotropy. However, the effect of the inclination angles was completely eliminated in the latter. It is considered that the complete non-oriented heating property relative to the magnetic flux direction allows the precise control of the ablation temperature during minimally invasive thermotherapy without a lead-wire connected to a fiber-optic thermometer. This newly designed Ti-coated device will be suitable for clinical use combined with its superior biocompatibility for ablation treatments using high-frequency induction heating.

## 1. Introduction

Recent advances in radiotherapy [1,2,3], chemotherapy [4,5,6], and endocrine therapy [7,8,9] have reduced the necessity of invasive surgical resections in human cancer treatments. Based on such background information, establishment of a more minimally invasive treatment has been desired for achievement of a favorable prognosis in patients suffering from cancers. In addition to the above-described treatments, thermotherapy has become a promising option to treat human solid cancers. In the case of a primary liver cancer, radio-frequency ablation (RFA) therapy utilizing the high-frequency current of 470 kHz has been widely carried out as a minimally invasive treatment [10,11,12,13]. However, RFA must be completed within a short time, because the patient must remain still while the high-frequency current flows from a needle electrode embedded in the tumor to grounding pads placed on the back or thigh of the patient. It has been reported that a tumor size greater than 30 mm in diameter is the main risk factor for local recurrence after RFA treatment [14,15]. Additionally, RFA can be safely performed in most cases; however, early or late complications related to mechanical or thermal damage may be observed during follow-up examinations [16]. Thus, it is essential to develop a more minimally invasive thermotherapy that could heat tumors at a lower temperature than RFA and also allows patients to slightly move during the treatments. 

In general, high-frequency induction heating is a process which is used to melt, bond, or harden metals and other electrically-conductive materials. This technique offers an attractive combination of fast and consistent heat for many manufacturing processes in the engineering field. Recently, we have focused on the application of this technique as a novel thermotherapy achieving more minimally invasive treatment in the medical field [17,18,19]. Thus, establishment of a novel ablation therapy which solves these problems is desired for the treatment of primary liver cancer. Although a ferromagnetic carbon steel rod is used as the main component of the ablation needle, encapsulation with a Ti layer is required to obtain a prominent biocompatibility in clinical use. In this thermotherapy, the pricking direction of the ablation needle seems to significantly vary due to the tumor location; therefore, a non-oriented heating property relative to the magnetic flux direction is indispensable for precise control of the treatment temperature. However, the shape magnetic anisotropy, which originates from the demagnetizing field coefficient, causes an undesirable effect on the heating property in the AC magnetic field [20].

In a previous study [21], we investigated the heating property of the Ti-coated carbon steel rod using a high-frequency output of 100 W (AC magnetic field: 1.69 A/m) at 300 kHz. According to experiments performed by changing the inner diameter of the Ti-tubes, there was an optimum thickness that minimized the effect of the inclination angles relative to the magnetic flux direction. Considering these results, we have attempted to fabricate a prototype ferromagnetic ablation needle, which is totally covered by the Ti layer having a superior biocompatibility [22,23]. The purpose of the present study was to evaluate its adaptability for a novel ablation cancer therapy utilizing high-frequency induction heating.

## 2. Materials and Methods

### 2.1. Materials

**Figure 1:**
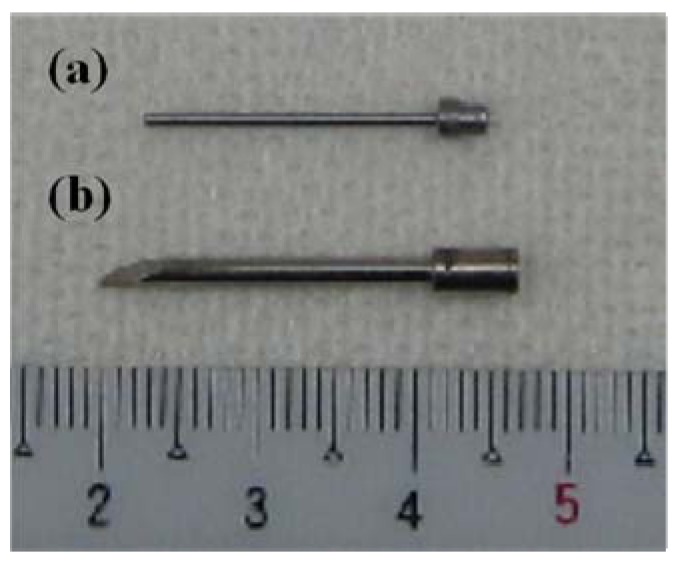
Appearance of the ferromagnetic carbon steel rod (**a**) and the newly designed prototype Ti-coated ablation needle used in the present study (**b**).

The ferromagnetic carbon steel rod containing 0.16% C used to generate heat in the AC magnetic field is shown in Figure 1a. While the full length of this carbon steel rod was 22.2 mm, its diameter was 1.0 mm over almost all of its 19.0 mm length. Additionally, the 3.2 mm long end possessed a larger diameter to some extent, corresponding to the gripping part of the ablation needle. The expanded end part possessed different diameters of 2.5 mm and 2.0 mm as seen in figure. Figure 1b shows the prototype Ti-coated ablation needle with a full length of 26.5 mm fabricated for subsequent animal experiments. The outer component having the Ti thickness of 0.4 mm was obtained from a Ti rod using a high precision CNC turning machine. The above-mentioned carbon steel rod was inserted into this outer component, and then a cover consisting of Ti was attached to the upper part by a press-fit insertion technique. Thus, this newly designed prototype ablation for the present study was completely encapsulated by a non-magnetic Ti layer. The outer diameter of the 19.0 mm long insertion part was determined to be 1.8 mm, so as to obtain the optimum Ti thickness of 0.4 mm in order to reduce the effect of the shape magnetic anisotropy [20,21]. The tip of the prototype ablation needle had an acute angle for easy insertion into the tumor, while a notch is made in the gripping part to fit the claw of Kocher’s forceps.

### 2.2. Experimental Procedure

**Figure 2:**
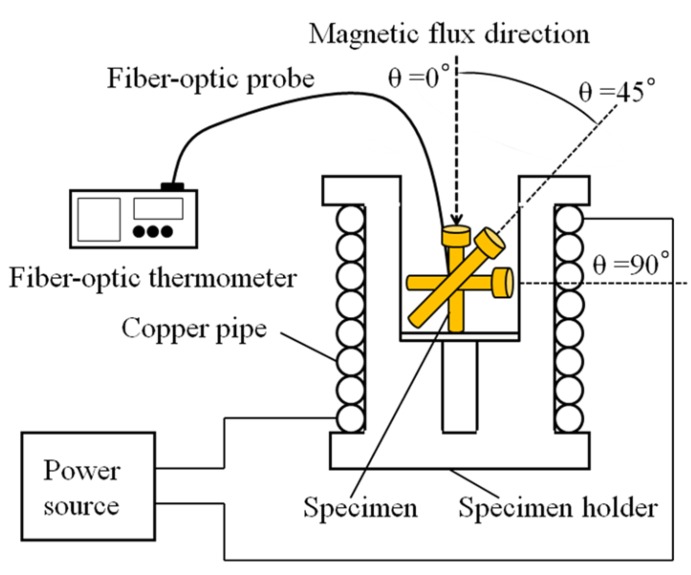
Setup for measuring the heating properties of the ferromagnetic carbon steel rod and the newly designed prototype ablation needle having different inclination angles relative to the magnetic flux direction.

Figure 2 shows the setup used for the measurement of the heating property in the AC magnetic field. Both the ferromagnetic carbon steel rod (Figure 1a) and the newly designed Ti-coated prototype ablation needle (Figure 1b) using the above carbon steel rod were placed in a high-frequency induction coil at the three different inclination angles of θ = 0°, 45° and 90° relative to the magnetic flux direction. The high-frequency induction coil was connected to a power supply through an impedance tuner. While the high-frequency outputs employed in the present study were 80 W, 90 W, and 100 W, these values corresponded to the AC magnetic fields of 1.40 kA/m, 1.50 kA/m, and 1.69 kA/m, respectively. The measured frequency was 300 kHz for all the specimens, and a fiber-optic thermometer was used to directly measure the increase in temperature (ΔT) in ambient air.

## 3. Results and Discussion

### 3.1. Heating Properties of the Ferromagnetic Mild Steel Rod

**Figure 3:**
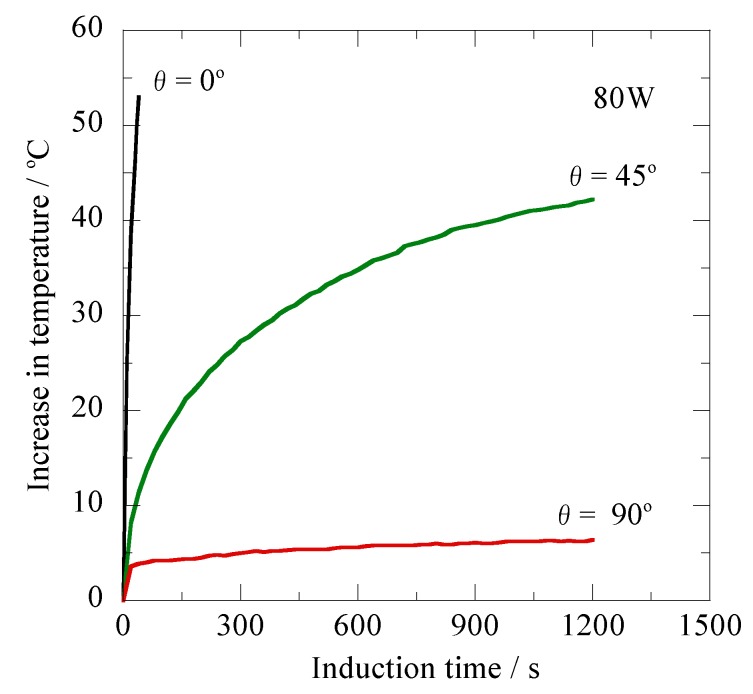
Changes in temperature of the ferromagnetic carbon steel rod having different inclination angles relative to the magnetic flux direction *versus* the induction time for the high-frequency output power of 80 W at 300 kHz.

Figure 3 shows the relationship between the increase in temperature (ΔT) and induction time for the ferromagnetic carbon steel rod (Figure 1a) with different inclination angles relative to the magnetic flux direction in the high-frequency output of 80 W (AC magnetic field: 1.40 kA/m) at 300 kHz. These results show the typical effect of the shape magnetic anisotropy on the heating property found in the rod-like ferromagnetic materials. The ΔT attains a markedly high value of 53.0 °C during the short time of 40 s in the θ = 0° specimen, whereas that of the θ = 90° specimen is as low as 6.4 °C even after the induction time of 1,200 s. For the θ = 45° specimen, the ΔT value continuously increased with the increasing induction time and reached 42.2 °C after the induction time of 1,200 s.

**Figure 4:**
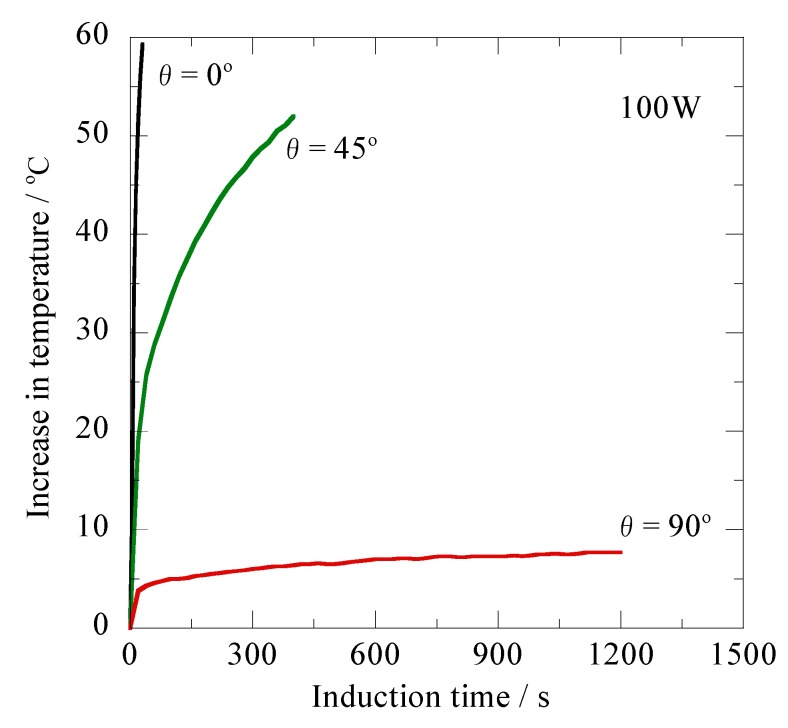
Changes in temperature of the ferromagnetic carbon steel rod having different inclination angles relative to the magnetic flux direction *versus* the induction time for thehigh-frequency output power of 100 W at 300 kHz.

Figure 4 shows the induction time dependence of the heating properties for the ferromagnetic carbon steel rod (Figure 1a) for the high-frequency output of 100W (AC magnetic field: 1.69 kA/m) at 300 kHz. As is obvious from the figure, both the θ = 0° and θ = 90° specimens possess almost the same temperature curves in comparison to the data shown in Figure 3. On the other hand, a significant increase in ΔT is found to occur for the θ = 45° specimen, and its value reached 52.0 °C after the induction time of 400 s. Because the various insertion angles into the tumors occur due to their location during clinical use, the direct use of the ferromagnetic carbon steel rod is unsuitable as the device for the novel ablation cancer therapy, in addition to its poor biocompatibility.

### 3.2. Heating Properties of the Prototype Ti-Coated Ablation Needle

**Figure 5:**
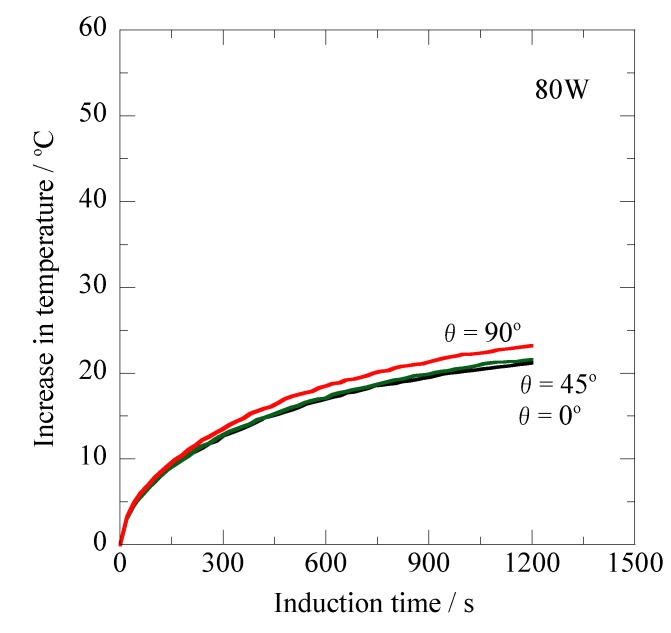
Changes in temperature of the prototype Ti-coated ablation needle at different inclination angles relative to the magnetic flux direction *versus* the induction time for the high-frequency output of 80 W at 300 kHz.

Figure 5 shows the relationship between the increase in temperature (ΔT) and induction time for the prototype Ti-coated ablation needle (Figure 1b) for the high-frequency output of 80 W (AC magnetic field: 1.40 kA/m) at 300 kHz. Both the θ = 0° and θ = 45° specimens exhibit overlapping temperature curves that reached the ΔT of about 21 °C after 1,200 s. The ΔT value similarly increased with the increasing induction time, and its value reached 23.2 °C after 1,200 s for the θ = 90° specimen. 

**Figure 6:**
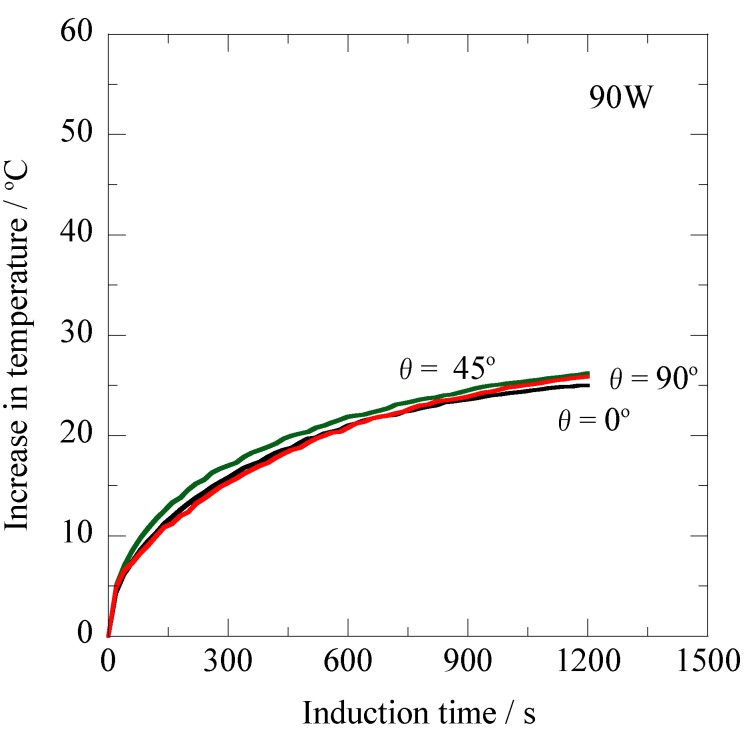
Changes in temperature of the prototype Ti-coated ablation needle at different inclination angles relative to the magnetic flux direction *versus* the induction time for the high-frequency output of 90 W at 300 kHz.

Figure 6 shows the induction time dependence of the heating property for the prototype Ti-coated ablation needle (Figure 1b) for the high-frequency output of 90 W (AC magnetic field: 1.50 kA/m) at 300 kHz. In this figure, it is noted that the three temperature curves are well overlapped *versus* the induction time. The ΔT values are approximately 26 °C after 1,200 s for all the specimens with the different inclination angles (θ = 0°, θ = 45° and θ = 90°) relative to the magnetic flux direction. The ΔT values after the induction time of 1200 s are slightly higher compared to the data given in Figure 5 as a result of the increase in the high-frequency output from 80 W to 90 W.

**Figure 7:**
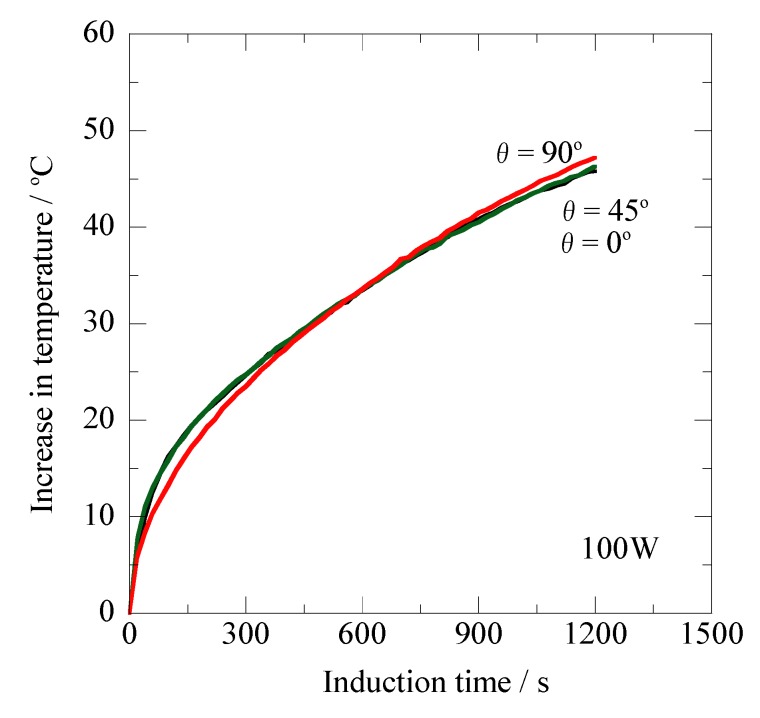
Changes in temperature of the prototype Ti-coated ablation needle at different inclination angles relative to the magnetic flux direction *versus* the induction time for the high-frequency output of 100 W at 300 kHz.

Figure 7 shows the induction time dependence of the heating property for the prototype Ti-coated ablation needle (Figure 1b) for the high-frequency output of 100 W (AC magnetic field: 1.69 kA/m) at 300 kHz. Also in this figure, the three temperature curves totally coincide with each other *versus* the induction time, reaching ΔT values of approximately 46 °C after 1,200 s.

### 3.3. Applicability of the Prototype Ti-Coated Needle for Ablation Cancer Therapy

Taking Figure 5, Figure 6, and Figure 7 into consideration, it is obvious that the non-oriented heating property relative to the magnetic flux direction is achieved with the prototype Ti-coated ablation needle fabricated in this study. For the Ti-tube involving the ferromagnetic carbon steel rod [21], the effect of the inclination angles resulting from the shape magnetic anisotropy [20] was significantly reduced by choosing the optimum Ti thickness; however, the complete non-oriented heating property has not been obtained because of the insufficient encapsulation by the non-magnetic Ti layer. It seems likely that the complete sealing of the ferromagnetic carbon steel rod plays a key role in achieving such a significant feature as the ablation needle for human cancer therapy. 

In the AC magnetic field, the hysteresis loss (P_h_) and eddy current loss (P_e_) remarkably affect the difference in the heating property of the ferromagnetic carbon steel rod. The Ti-coated carbon steel rod with a small Ti thickness presumably possesses a higher maximum magnetic flux density resulting in the high P_h_ and P_e_ values. However, the increase in the Ti thickness produces a decrease in the cross-sectional area of the embedded carbon steel rod. The low amount of magnetic flux passing through this reduced area leads to the lower effect of P_h_ and P_e_ on the ΔT value. It seems likely that the balanced effects of P_h_ and P_e_ play an important role in achieving the non-oriented heating property. In addition, the magnetic penetration depth (δ) is closely associated with the thermal properties of the Ti-coated carbon steel rod based on the electrical resistivity and relative magnetic permeability. Considering these factors, there is an optimum condition to obtain a non-oriented heating property resulting from the combined effects of P_h_, P_e_ and δ. In conclusion, the effect of the shape magnetic anisotropy is drastically reduced using a carbon steel rod surrounding by the Ti layer with an optimum thickness. 

Comparing Figure 7 with Figure 4, a remarkable difference is observed in the heating properties between the ferromagnetic carbon steel rod and the prototype Ti-coated ablation needle. As is obvious from Figure 7, the temperature curves of the θ = 0° and θ = 45° specimens remarkably lower their slope during the induction time of 1,200 s. These results are attributable to the significantly reduced effect of the shape magnetic anisotropy explained in terms of the demagnetization coefficient [24]. On the other hand, the drastic ΔT enhancement, which reached approximately 46 °C after 1,200 s, is observed in the temperature curve of the θ = 90° specimen, even though the ΔT value is at most 7.7 °C after 1,200 s in the ferromagnetic carbon steel rod shown in Figure 4. It is important to note that a fairly enlarged area perpendicular to the magnetic flux direction is achieved in the high-frequency induction coil for the θ = 90° specimen. The complete encapsulation provides an uninterrupted closed circuit for the Ti layer surrounding the enlarged area of the ferromagnetic carbon steel rod. The eddy current flowing near the surface of the Ti layer significantly affects the ΔT enhancement, thus causing overlapping of the temperature curves of the θ = 0° and θ = 45° specimens.

In clinical use, it is essential to strictly control the ablation temperature so as not to exceed the optimum temperature. The ΔT value of more than 40 °C seems to be undesirable for the ablation treatments, because it produces a remarkably deleterious effect on the normal cells surrounding the tumors. It is considered from Figure 7 that the high-frequency output of 100 W results in the excessive ΔT enhancement. The prototype Ti-coated ablation needle enables us to indirectly control the treatment temperature by setting the optimized high-frequency output and treatment time. It is particularly emphasized that the novel thermotherapy will be conveniently carried out utilizing the non-oriented Ti-coated ablation needle, because there is no necessity to use a lead-wire connected to the fiber-optic thermometer during the treatment. This prototype ablation needle having a superior biocompatibility [22,23] will be employed in subsequent animal experiments in order to certify its clinical applicability.

## 4. Conclusions

The heating properties of a ferromagnetic carbon steel rod and a prototype Ti-coated ablation needle were investigated in several high-frequency outputs ranging from 80 W to 100 W with the aim of studying its applicability as a novel ablation cancer therapy. In the experiments, by changing the inclination angles (θ = 0°, θ = 45° and θ = 90°) relative to the magnetic flux direction, the former exhibited drastically different heating properties among three inclination angles due to the effect of the shape magnetic anisotropy. However, the latter had a complete non-oriented heating property relative to the magnetic flux direction, suggesting that precise control of the ablation temperature is possible during clinical use without a lead-wire connected to a fiber-optic thermometer.
